# Preoperative Predictors of Lymph Node Metastasis in Colon Cancer

**DOI:** 10.3389/fonc.2021.667477

**Published:** 2021-05-31

**Authors:** Yansong Xu, Yi Chen, Chenyan Long, Huage Zhong, Fangfang Liang, Ling-xu Huang, Chuanyi Wei, Shaolong Lu, Weizhong Tang

**Affiliations:** ^1^ Department of Emergency, The First Affiliated Hospital of Guangxi Medical University, Nanning, China; ^2^ Guangxi Clinical Research Center for CRC, Department of Gastrointestinal Surgery, Guangxi Medical University Cancer Hospital, Nanning, China; ^3^ Department of Anorectal Surgery, Zhuzhou Center Hospital, Zhuzhou, China; ^4^ Department of Medical Oncology, The First Affiliated Hospital of Guangxi Medical University, Nanning, China; ^5^ Department of Hepatological Surgery, Guangxi Medical University Cancer Hospital, Nanning, China

**Keywords:** colon cancer, biomarkers, lymph node metastasis, nomogram, diagnosis

## Abstract

**Background:**

Lymph node metastasis (LNM) is a well-established prognostic factor for colon cancer. Preoperative LNM evaluation is relevant for planning colon cancer treatment. The aim of this study was to construct and evaluate a nomogram for predicting LNM in primary colon cancer according to pathological features.

**Patients and Methods:**

Six-hundred patients with clinicopathologically confirmed colon cancer (481 cases in the training set and 119 cases in the validation set) were enrolled in the Affiliated Cancer Hospital of Guangxi Medical University from January 2010 to December 2019. The expression of molecular markers (p53 and β-catenin) was determined by immunohistochemistry. Multivariate logistic regression was used to screen out independent risk factors, and a nomogram was established. The accuracy and discriminability of the nomogram were evaluated by consistency index and calibration curve.

**Results:**

Univariate logistic analysis revealed that LNM in colon cancer is significantly correlated (P <0.05) with tumor size, grading, stage, preoperative carcinoembryonic antigen (CEA) level, and peripheral nerve infiltration (PNI). Multivariate logistic regression analysis confirmed that CEA, grading, and PNI were independent prognostic factors of LNM (P <0.05). The nomogram for predicting LNM risk showed acceptable consistency and calibration capability in the training and validation sets.

**Conclusions:**

Preoperative CEA level, grading, and PNI were independent risk factor for LNM. Based on the present parameters, the constructed prediction model of LNM has potential application value.

## Introduction

Colorectal cancer (CRC) is one of the most common malignancies with the second highest death rate in 2018 (1, 2). Doctors can formulate a variety of treatment plans for CRC because of the continuous enrichment of treatment options. However, surgical treatment remains the primary treatment for CRC. The rate of lymph node metastasis (LNM) in early CRC is 6.9–19.6% (3–6). The scope of surgery can be adopted for patients with CRC to avoid excessive treatment. Preoperative evaluation of LNM risk in colon cancer may help i) to provide information on an important prognostic factor (7, 8) and ii) to plan the most appropriate therapeutic and staging strategies, particularly in the neoadjuvant setting  (9).

LNM in colon cancer can be predicted by histopathological markers. Tumor stage and grade are well-recognized predictors (10–12). However, in clinical practice, statistical results cannot be directly applied to individual clinical diagnosis and treatment. Although a nomogram model for colon cancer LNM has been developed for clinical use, the validation of the model with external data sets is still lacking (8, 10). Therefore, we retrospectively analyzed the data of 600 patients with primary colon cancer admitted in 2010 and 2019 and attempted to establish a nomogram prediction model for colon cancer.

## Methods

### Study Selection

**Figure 1:**
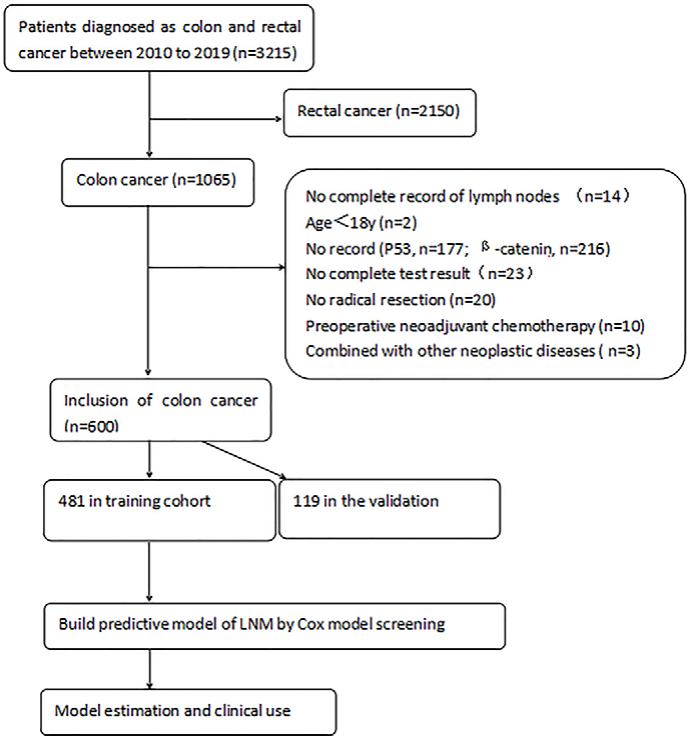
Data screening process.

This study retrospectively collected 600 clinical cases of colon cancer confirmed by postoperative pathology in the Affiliated Cancer Hospital of Guangxi Medical University from January 2010 to December 2019. The cases included 369 males and 231 females (age range, 19–87 years; mean age, 60 years old). We defined the training and the validation groups by time in the study. The training set was used to establish the model, and the validation set was used to verify the performance of the model. The training group was composed of 481 patients who were admitted between January 2013 and December 2019, and the validation group consisted of 119 patients who were hospitalized between January 2010 and December 2012. The inclusion criteria were as follows (1): colon cancer was confirmed by pathological examination (2); lymph node dissection was performed, and the number of lymph nodes detected was at least 12 (3); radical surgery was performed (4); complete clinical data and pathological information were available (5); all patients underwent lymph node D3 dissection (D3 lymph node dissection refers to the dissection of parenteral, intermediate, and central lymph nodes); and (6) the postoperative pathological diagnosis was colorectal adenocarcinoma. The exclusion criteria were as follows (1): preoperative neoadjuvant chemotherapy was performed; and (2) the co-occurrence of other tumor diseases at the time of diagnosis (**Figure 1**). The surgery was performed by an experienced associate chief physician or a chief physician who is able to perform laparoscopic surgery independently.

### Variable Analysis

The variables selected in this study included the following clinicopathologic data and biomarkers: gender, age, BMI, drinking status, smoking status, tumor site, tumor size, carcinoembryonic antigen (CEA) level, platelet–lymphocyte ratio (PLR), neutrophil–lymphocyte ratio (NLR), LNM, pathological tumor (pT) stage, tumor differentiation, peripheral nerve infiltration (PNI), p53 expression, vascular infiltration, and β-catenin expression. The optimal cutoff value (PLR = 279, NLR = 4.24, tumor diameter = 3.35), sensitivity, and specificity were calculated according to the receiver operating characteristic (ROC) curve. The preoperative CEA value was 5 U/ml. Immunohistochemical staining score is based on the proportion of stained tumor cells. The protein expression levels of p53 and β-catenin were divided into high expression (>20%) and low expression (≤20%).

### Statistical Analysis

In this study, SPSS 26.0 and R software (version 3.6.1, www.r-project.org) were used in statistical analyses. P values were calculated by Chi-Square test for categorical variables. P <0.05 was considered statistically significant. We used Cox proportional risk model to determine the independent factors that affect LNM based on the variables selected in the univariate analysis. We used logistic regression model in multivariate analysis to predict LNM. Consistency index (C-index) was calculated, and the calibration results were evaluated by calibration curves. The nomogram was drawn by referring to the step-by-step method provided by Zhang et al. (13). We used a series of software packages in R, including rmda, proc, foreign, nrichens, rms, and survival, to build the nomogram.

## Results

### Patient Characteristics

The parameters of the training and verification sets are shown in **Table 1**. The training set consisted of 302 males and 179 females. The average age was 60 years. Among them, 398 cases had high P53 expression and 83 cases had low P53 expression. The validation group consisted of 78 men and 41 women. Among them, 95 cases had high p53 expression and 24 cases had low p53 expression. LNM was associated with the following clinicopathological parameters: tumor differentiation (P = 0.004), CEA (P ≤0.01), pT stage (P = 0.004), PNI (P ≤0.01), vascular infiltration (P ≤0.01), and tumor diameter (P = 0.033, **Table 2**).

**Table 1:** Clinicopathological characteristics of colon cancer patients in two data sets.

Variables		Training (481)	Validation (119)
Gender	Male	353	78
	Female	128	41
Age	<60	243	70
	≥60	238	49
BMI	<24	323	74
	≥24	153	45
Drinking	Never	285	60
	Ever	196	59
Smoking	Never	200	47
	Ever	281	72
Tumor site	Left	255	55
	Right	230	64
Maximum tumor diameter	<3.35	244	67
	≥3.35	237	52
Grading	Low	101	20
	Moderate	235	54
	High	145	45
pT stage	T1/2	15	9
	T3	53	10
	T4	413	100
Pre-CEA	<5	211	54
	≥5	270	65
Pre-PLR	<279	221	54
	≥279	260	65
Pre-NLR	<4.24	221	54
	≥4.24	260	65
P53 expression	Low/no	83	24
	High	398	95
β-catenin expression	Low/no	128	29
	High	353	90
Vascular invasion	Present	174	60
	Absent	307	59
PNI	Present	272	80
	Absent	209	39

BMI, Body mass index; CEA, Carcinoembryonic antigen; PLR, Platelet/lymphocyte; NLR, Neutrophil/lymphocyte; p-T, pathological Tumor Stage; PNI, Peripheral nerve infiltration.

**Table 2:** Relationship between lymph node metastasis and clinicopathology in training set.

		LNM (+)	LNM (–)	*P*-value
Sex	male	127	175	0.162
	female	87	93	
Age	<60	133	180	0.328
	≥60	81	88	
BMI	<24	122	165	0.452
	≥24	92	103	
Drinking	Never	133	178	0.356
	Ever	81	90	
Smoking	Never	120	165	0.306
	Ever	94	103	
Grading	low	40	26	0.004
	moderate	170	227	
	high	4	17	
Pre-PLR	<279	166	218	0.268
	<279	48	49	
Pre-NLR	<4.25	169	229	0.050
	≧4.25	45	38	
Pre-CEA	<5	88	176	0.000
	≧5	126	91	
pT stage	1/2	16	46	0.004
	3	80	99	
	4	118	122	
Tumor site	left	109	135	0.935
	right	105	132	
Vascular invasion	absent	99	206	0.000
	present	115	61	
PNI	absent	69	140	0.000
	present	145	127	
β-catenin expression	Low/no	65	77	0.714
	High	149	190	
P53 expression	Low/no	29	54	0.054
	High	185	213	
Maximum tumor diameter	<3.75	33	62	0.033
	≧3.75	181	205	

BMI, Body mass index; CEA, Carcinoembryonic antigen; PLR, Platelet/lymphocyte; NLR, Neutrophil/lymphocyte; p-T, pathological Tumor Stage; PNI, Peripheral nerve infiltration; LNM, Lymph Node Metastasis.

### Univariate and Multivariate Analyses of Clinical Variables

Univariate logistic regression analysis showed that colon cancer LNM was correlated with tumor size, grading, pT stage, preoperative CEA level, and PNI (P <0.05, **Table 2**). Multivariate logistic regression analysis showed that preoperative CEA, grading, and PNI were correlated with LNM in colon cancer (P <0.05). PNI was an independent predictor of LNM in colon cancer (P <0.05, **Table 3**).

**Table 3:** Logistic analysis between clinical and pathological parameters and LNM in training set.

Variables		Univariate analysis		Multivariate analysis	
		95%CI	*P*	95%CI	*P*
Gender	Male	–	0.163		
	Female	1.303 (0.899–1.890)			
Age, mean	60	1.005 (0.991–1.019)	0.477		
BMI	<24	–	0.354		
	≥24	0.756 (0.428–1.322)			
Drinking	Never	–	0.452		
	Ever	0.485 (0.188–0.651)			
Smoking	Never	–	0.867		
	Ever	0.185 (0.265–1.124)			
Tumor site	Left		0.935		
	Right	0.985 (0.688–1.412)			
Maximum tumor diameter	<3.35	–	0.034		0.478
	≥3.35	1.659 (1.040–2.647)		1.203 (0.722–2.005)	
Grading	Low	–	0.005		*0.016*
	Moderate	0.487 (0.286–0.829)		0.529 (0.303–0.923)	
	High	0.186 (0.055–0.627)		0.333 (0.095–1.165)	
pT stage	T1/2	–	0.006		0.243
	T3	2.323 (1.224–4.409)		1.526 (0.768–3.034)	
	T4	2.781 (1.492–5.183)		1.554 (0.789–3.060)	
Pre-CEA	<5	–	≤0.001		≤0.001
	≥5	2.769 (1.910–4.016)		2.673 (1.823–3.920)	
Pre-PLR	<279	–	0.269		
	≥279	1.286 (0.823–2.010)			
Pre-NLR	<4.24	–	0.051		
	≥4.24	1.605 (0.998–2.581)			
P53 expression	Low/no	–	0.056		
	High	1.617 (0.988–2.646)			
β-catenin expression	Low/no	–	0.714		
	High	0.929 (0.627–1.377)			
Vascular invasion	Absent	–			0.325
	Present	0.255 (0.172–0.377)	0.016	0.355 (0.193–0.558)	
PNI	Absent	–	≤0.001		≤0.001
	Present	2.317 (1.594–3.367)		2.249 (1.524–3.318)	

BMI, Body mass index; CEA, Carcinoembryonic antigen; PLR, Platelet/lymphocyte; NLR, Neutrophil/lymphocyte; p-T, pathological Tumor Stage; PN, Peripheral nerve infiltration.

### Construction and Validation of the Nomogram

**Figure 2:**
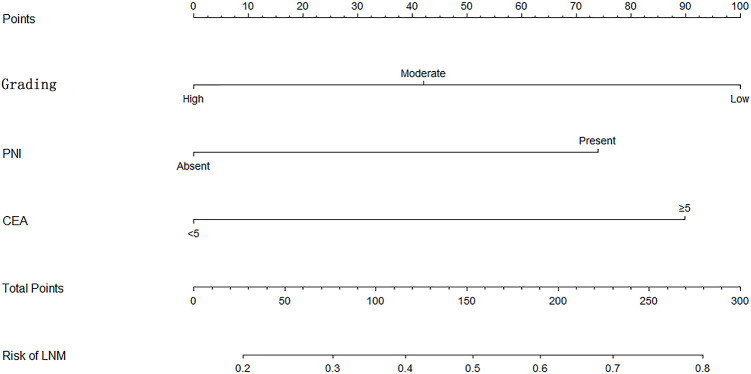
Nomogram constructed according to clinicopathological parameters.

**Figure 3:**
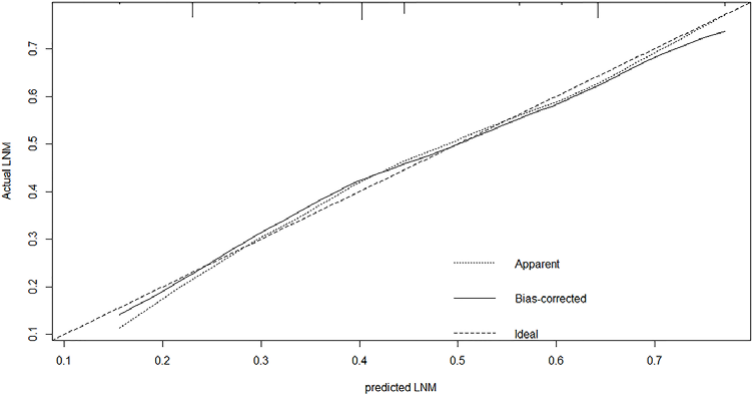
The calibration plot showed a high fit between actual and predicted lymph node metastases in training set.

**Figure 4:**
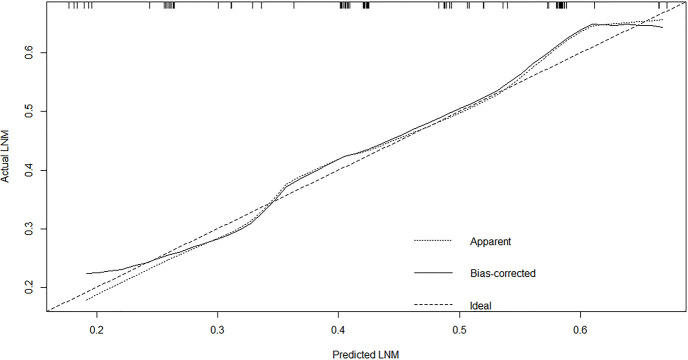
The calibration plot showed a high fit between actual and predicted lymph node metastases in external set.

**Figure 5:**
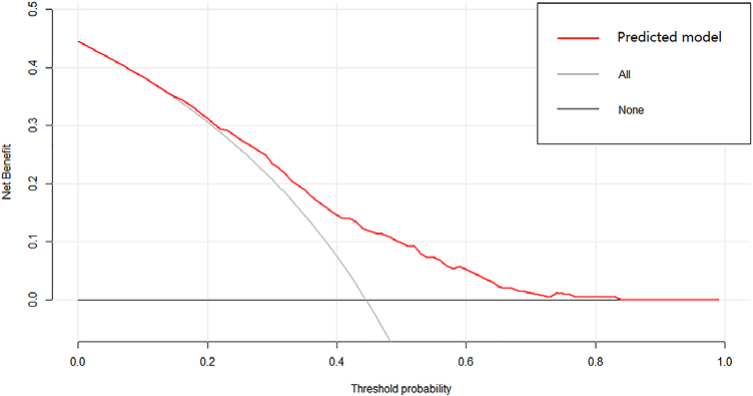
Decision curve analysis showed that patients had a good net benefit from this model.

A nomogram was established to predict the risk of LNM in colon cancer (**Figure 2**). The C-index was 0.686 in the training set and 0.644 in the validation set. The bootstrap method was internally and externally validated to show the good fit of the model. The prediction of LNM risk highly fits the actual metastasis (**Figures 3**, **4**). Declining curve analysis (DCA) shows that the net benefit rate of the model is in the range of 0.2–0.8 for pT, which is higher than that in the limit curve (**Figure 5**), using the nomogram. The corresponding score was determined according to the clinicopathological data of the patient and the tumor. All the points were added to obtain the total. Finally, the risk value that corresponds to the total score was determined. For example, the following results were obtained: preoperative CEA >5 U/ml; the total score of moderate differentiation with PNI in patients with colon cancer was 90 + 42 + 75 = 207; then, the corresponding risk for LNM was 65%. Physicians and patients can use the nomogram to predict the risk of LNM and individually assess patients more accurately to help them choose a more appropriate treatment plan.

## Discussion

In this study, a clinical model for the individualized prediction of LNM in colon cancer was established. The model consists of basic data and clinical risk factors. Sixteen clinicopathological features were analyzed by univariate regression. Three clinical and pathological risk indicators were selected as independent risk factors for multivariate logistic analysis. Finally, the independent risk factors were applied to establish a visual prediction model. We analyzed an external validation set, which did not involve those enrolled in the internal data set, to further validate the predictive performance of the model. The model has promising clinical value in predicting LNM. The results of this study indicated that the LNM-associated nomogram has a favorable application prospect in patients with colon cancer. Similar to previous reports (3, 14–17), this study found that tumor differentiation, preoperative CEA level, and PNI were independent risk factors associated with LNM in colon cancer.

Among the three potential clinical risk factors, CEA level is the earliest clinical indicator and closely related to LNM in colon cancer (14). Several studies have examined the number, distribution, size, and percentage of lymph nodes involved to assess colon cancer survival (18–20). A multicenter study found that higher CEA level and worse tumor differentiation leads to more LNM (16). Han et al. also suggested that LNM and even the distant metastasis of colon cancer are positively correlated with CEA level (21). We found by analyzing the nomogram constructed in this study that, except for tissue differentiation, preoperative CEA level had the greatest influence on the model among all the potential risk factors. The results of this study further support the idea that patients with a higher preoperative CEA level should be considered at high risk for colon cancer LNM. Martin R. Weiser and colleagues developed a colon cancer recurrence nomogram to predict relapse based on the number of positive and negative lymph nodes, lymphovascular invasion, and other risk factors (22). Compared with the vascular infiltration of CRC, few studies have been conducted on the correlation between PNI and LNM in colon cancer. Although PNI can be considered a way of local diffusion, it can also be the only way to determine the range of distant metastasis (23). The number of LNM in patients with PNI is twice than that in patients without PNI (24). Compared with the 12.6–30% incidence of PNI reported in other studies (10, 24, 25), the present study found that the event rate was nearly 50%, which may lead to a high incidence that differed from the inclusion criteria. The study included patients who underwent radical resection of colon cancer. The rate of LNM in patients with PNI was 53.3% (145/272), which was remarkably higher than that in patients without PNI (69/209). Studies have found statistically substantial differences in the expression of P53 and β-catenin in CRC LNM. P53 overexpression or decreased β-catenin expression is more common in patients with LNM from CRC (26, 27). In addition, disease-free survival is reduced in patients with p53 overexpression (28). Although the above relationship was not found in the present study, we still cannot ignore the potential role of P53 and β-catenin in LNM.

Regional LNM prediction models for CRC had been proposed to better apply the research results to clinical work. However, these models were developed for CRC. Specific prediction models for LNM in colon cancer are lacking. Two scholars constructed a risk model of LNM in T1 stage CRC using clinical and pathological parameters (11, 29). Clinical imaging nomograms based on radiomics and clinical risk factors were constructed to improve the predictive power of preoperative LNM (30, 31). The accuracy of the model is better than that of simple clinical or pathological parameters. Many scholars agree that the model can be used for the preoperative prediction of LNM in patients with CRC and other tumors (11, 32, 33). The nomogram’s area under the ROC curve (AUC) for predicting LNM of non-digestive tract tumors using clinicopathological parameters was above 0.75, which was remarkably higher than that for digestive tract tumors, including colon cancer (34–36). Nomogram is widely used in CRC because of its remarkability for individual treatment and prognostic prediction. The C-index showed good discrimination in the internal (C = 0.687) and external validation sets (C = 0.644). The nomogram’s calibration curve is very close to the optimal curve. Doctors can add scores corresponding to each index state to obtain the total score in clinical application and then obtain the corresponding LNM probability because of the model’s convenience, economy, and practicality. The application of this nomogram will contribute to an accurate understanding of the disease and help doctors and patients choose a personalized treatment. After treatment, the nomogram could help doctors to distinguish high- and low-risk patients and formulate follow-up for high-risk patients. Some adjuvant treatments, such as chemical drugs and targeted drugs, should be given to consolidate therapeutic effects and help postoperative patients, especially high-risk patients. The nomogram is a useful clinical tool that can reduce colectomy after endoscopic resection among patients with T1 colon cancer. The effectiveness of conventional diagnostic methods is usually determined by creating ROC curves and calculating the AUC. However, ROC only considers the specificity and sensitivity of the method and pursues accuracy. We speculated whether the nomogram is clinically accurate enough and whether patients benefit from the use of the nomogram. Therefore, in addition to building the model, we used DCA to determine whether patients would benefit from the clinical prediction model. The DCA results show that the net benefit rate of the model was in the range of 0.2–0.8 for pT, which was higher than that in the extreme curve.

This study has the following limitations. 1. This study is a retrospective study, and selectivity bias is inevitable. 2. Data from a single center at different periods were used for the external validation of the model. Thus, the promotion and use of the model should be performed cautiously. 3. Regional lymph nodes were not subdivided and compared. 4. The model reflects the basic situation of the patient at a specific time. The use span of the model may show certain limitations with changes in time. 5. Validation of external data from multiple medical centers is also needed to promote the model. Therefore, the next research focus is to test the model with external data. In addition, we considered the influence of PNI and tissue differentiation degree on CRC when setting possible influencing factors. Although the grading and PNI can be obtained preoperatively through a puncture, this procedure often requires an adequate amount of tissue and therefore needs to be performed with caution.

## Conclusion

Based on tumor differentiation, preoperative CEA level and PNI, a nomogram model was established for predicting the incidence of LNM in colon cancer patients. The predicted model of LNM risk of colon cancer has potential application value. However, further prospective studies with large samples are still necessary.

## Data Availability Statement

The raw data supporting the conclusions of this article will be made available by the authors, without undue reservation.

## Ethics Statement

The studies involving human participants were reviewed and approved by The First Affiliated Hospital of Guangxi Medical University. The patients/participants provided their written informed consent to participate in this study.

## Author Contributions

YX, YC, and CL contributed equally to this study. YX designed the experiments and wrote the manuscript. L-xH, CW, and SL collected the date. YC and SL analyzed the data. WT funded the project. YC, CL, FL, and HZ checked and revised the manuscript and confirmed all the data in the manuscript. All authors contributed to the article and approved the submitted version.

## Funding

This work was supported by (1): National Natural Science Foundation of China (81973533) (2), 2019 Guangxi University High-level Innovation Team and the Project of Outstanding Scholars Program, and (3) Guangxi Science and Technology Project (2019AC03004 and AD19245197).

## Conflict of Interest

The authors declare that the research was conducted in the absence of any commercial or financial relationships that could be construed as a potential conflict of interest.
